# 3-Anilino-1-(isopropyl­amino)­propan-2-ol

**DOI:** 10.1107/S1600536812013256

**Published:** 2012-03-31

**Authors:** Xuehui Hou, Ping Hu, Quanjian Lv

**Affiliations:** a Department of Quality Detection and Management, Zhengzhou College of Animal Husbandry Engineering, Zhengzhou 450011, People’s Republic of China

## Abstract

The title compound, C_12_H_20_N_2_O, was obtained by the reaction of *N*-(oxiran-2-ylmeth­yl)aniline and propan-2-amine. In the crystal, mol­ecules are linked by O—H⋯N and N—H⋯O hydrogen bonds into chains parallel to the *b* axis.

## Related literature

For applications of the amino alcohols and their derivatives, see: Ellison & Gandhi (2005 ▶); Li *et al.* (2004 ▶).
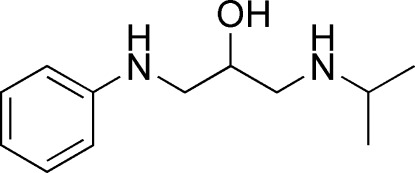


## Experimental

### 

#### Crystal data

C_12_H_20_N_2_O
*M* *_r_* = 208.30
Monoclinic, 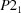

*a* = 8.7676 (8) Å
*b* = 6.4662 (6) Å
*c* = 11.1677 (12) Å
β = 105.290 (1)°
*V* = 610.72 (10) Å^3^
*Z* = 2
Mo *K*α radiation
μ = 0.07 mm^−1^
*T* = 296 K
0.50 × 0.49 × 0.40 mm

#### Data collection

Siemens SMART CCD area-detector diffractometer
Absorption correction: multi-scan (*SADABS*; Sheldrick, 1996 ▶) *T*_min_ = 0.965, *T*_max_ = 0.971
3608 measured reflections
1449 independent reflections
990 reflections with *I* > 2σ(*I*)
*R*_int_ = 0.049

#### Refinement

*R*[*F* ^2^ > 2σ(*F* ^2^)] = 0.046
*wR*(*F* ^2^) = 0.116
*S* = 0.94
1449 reflections
150 parameters
1 restraint
H atoms treated by a mixture of independent and constrained refinement
Δρ_max_ = 0.18 e Å^−3^
Δρ_min_ = −0.13 e Å^−3^

### 

Data collection: *SMART* (Siemens, 1996 ▶); cell refinement: *SAINT* (Siemens, 1996 ▶); data reduction: *SAINT*; program(s) used to solve structure: *SHELXS97* (Sheldrick, 2008 ▶); program(s) used to refine structure: *SHELXL97* (Sheldrick, 2008 ▶); molecular graphics: *SHELXTL* (Sheldrick, 2008 ▶); software used to prepare material for publication: *SHELXTL*.

## Supplementary Material

Crystal structure: contains datablock(s) I, global. DOI: 10.1107/S1600536812013256/rz2719sup1.cif

Structure factors: contains datablock(s) I. DOI: 10.1107/S1600536812013256/rz2719Isup2.hkl

Supplementary material file. DOI: 10.1107/S1600536812013256/rz2719Isup3.cml

Additional supplementary materials:  crystallographic information; 3D view; checkCIF report

## Figures and Tables

**Table 1:** Hydrogen-bond geometry (Å, °)

*D*—H⋯*A*	*D*—H	H⋯*A*	*D*⋯*A*	*D*—H⋯*A*
O1—H1*O*⋯N1^i^	0.79 (4)	2.09 (4)	2.878 (3)	173 (3)
N2—H2*N*⋯O1^i^	0.89 (3)	2.26 (3)	3.141 (3)	170 (3)

Symmetry code: (i) 
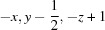
.

**Fig. 1.**
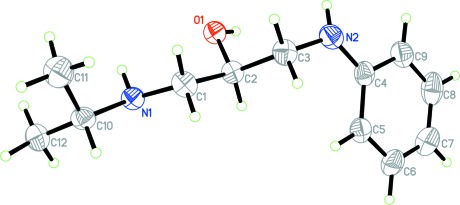
The molecular structure of the compound, with atom labels and 50% probability displacement ellipsoids.

**Fig. 2.**
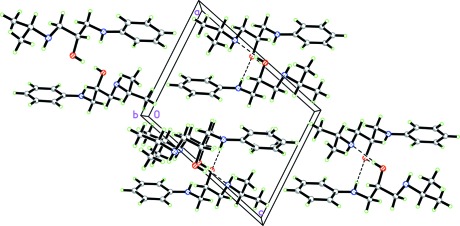
Crystal packing of the title compound viewed along the b axis, showing the molecular chains formed by intermolecular hydrogen bonds (dashed lines).

## supplementary crystallographic information

### Comment

Amino alcohols are important structural elements for asymmetric catalysis (Li *et al.*, 2004) as well as in biologically active compounds (Ellison & Gandhi, 2005). In order to develop new applications for amino alcohols and their derivatives, structural modifications of these compounds have been extensively investigated. As a contribution in this field, we report here the crystal structure of the title compound.

The molecular structure of the title compound is shown in Fig. 1. A l l bond lengths and angles are not unexceptional. In the crystal structure (Fig. 2), intermolecular O—H···N and N—H···O hydrogen bonds (Table 1) link molecules into chains running parallel to the *b* axis.

### Experimental

To a solution of *N*-(oxiran-2-ylmethyl)aniline (14.9 g, 0.1 mol) in acetone (200 ml), propan-2-amine (86.7 ml, 1.0 mol) was added. The mixture was stirred at room temperature for 6 h, then it was concentrated under reduced pressure and purified by crystallization from ethyl acetate, giving colourless single crystals of the title compound suitablu for X-ray analysis.

### Refinement

The amine and hydroxy H atoms were located in a difference Fourier map and refined freely. All other H atoms were placed geometrically and treated as riding on their parent atoms, with C—H = 0.93–0.96 Å and *U*_iso_(H) = 1.2*U*_eq_(C) or 1.5*U*_eq_(C) for methyl H atoms. In the absence of significant anomalous scattering, 464 Friedel pairs were merged.

### Crystal data

**Table:**

C_12_H_20_N_2_O	*F*(000) = 228
*M**_r_* = 208.30	*D*_x_ = 1.133 Mg m^−^^3^
Monoclinic, *P*2_1_	Mo *K*α radiation, λ = 0.71073 Å
Hall symbol: P 2yb	Cell parameters from 1130 reflections
*a* = 8.7676 (8) Å	θ = 3.4–22.5°
*b* = 6.4662 (6) Å	µ = 0.07 mm^−^^1^
*c* = 11.1677 (12) Å	*T* = 296 K
β = 105.290 (1)°	Block, colourless
*V* = 610.72 (10) Å^3^	0.50 × 0.49 × 0.40 mm
*Z* = 2	

### Data collection

**Table:**

Siemens SMART CCD area-detector diffractometer	1449 independent reflections
Radiation source: fine-focus sealed tube	990 reflections with *I* > 2σ(*I*)
Graphite monochromator	*R*_int_ = 0.049
phi and ω scans	θ_max_ = 27.0°, θ_min_ = 1.9°
Absorption correction: multi-scan (*SADABS*; Sheldrick, 1996)	*h* = −10→11
*T*_min_ = 0.965, *T*_max_ = 0.971	*k* = −8→4
3608 measured reflections	*l* = −13→14

### Refinement

**Table:**

Refinement on *F*^2^	Primary atom site location: structure-invariant direct methods
Least-squares matrix: full	Secondary atom site location: difference Fourier map
*R*[*F*^2^ > 2σ(*F*^2^)] = 0.046	Hydrogen site location: inferred from neighbouring sites
*wR*(*F*^2^) = 0.116	H atoms treated by a mixture of independent and constrained refinement
*S* = 0.94	*w* = 1/[σ^2^(*F*_o_^2^) + (0.0624*P*)^2^] where *P* = (*F*_o_^2^ + 2*F*_c_^2^)/3
1449 reflections	(Δ/σ)_max_ < 0.001
150 parameters	Δρ_max_ = 0.18 e Å^−^^3^
1 restraint	Δρ_min_ = −0.13 e Å^−^^3^

### Special details

**Table:**

Geometry. All e.s.d.'s (except the e.s.d. in the dihedral angle between two l.s. planes) are estimated using the full covariance matrix. The cell e.s.d.'s are taken into account individually in the estimation of e.s.d.'s in distances, angles and torsion angles; correlations between e.s.d.'s in cell parameters are only used when they are defined by crystal symmetry. An approximate (isotropic) treatment of cell e.s.d.'s is used for estimating e.s.d.'s involving l.s. planes.
Refinement. Refinement of *F*^2^ against ALL reflections. The weighted *R*-factor *wR* and goodness of fit *S* are based on *F*^2^, conventional *R*-factors *R* are based on *F*, with *F* set to zero for negative *F*^2^. The threshold expression of *F*^2^ > σ(*F*^2^) is used only for calculating *R*-factors(gt) *etc*. and is not relevant to the choice of reflections for refinement. *R*-factors based on *F*^2^ are statistically about twice as large as those based on *F*, and *R*- factors based on ALL data will be even larger.

### Fractional atomic coordinates and isotropic or equivalent isotropic displacement parameters (Å^2^)

**Table:**

	*x*	*y*	*z*	*U*_iso_*/*U*_eq_	
N1	−0.0180 (3)	0.3886 (4)	0.30241 (18)	0.0486 (6)	
N2	0.2976 (3)	−0.1975 (4)	0.5341 (2)	0.0607 (7)	
O1	−0.00479 (19)	0.0345 (3)	0.4500 (2)	0.0517 (5)	
C1	0.1036 (3)	0.2296 (5)	0.3115 (2)	0.0567 (8)	
H1A	0.1998	0.2945	0.3028	0.068*	
H1B	0.0686	0.1324	0.2437	0.068*	
C2	0.1394 (3)	0.1128 (4)	0.4333 (2)	0.0478 (7)	
H2	0.1871	0.2073	0.5015	0.057*	
C3	0.2559 (3)	−0.0607 (5)	0.4288 (2)	0.0571 (8)	
H3A	0.2115	−0.1434	0.3554	0.069*	
H3B	0.3523	0.0016	0.4185	0.069*	
C4	0.4017 (3)	−0.1471 (5)	0.6467 (2)	0.0531 (7)	
C5	0.4858 (3)	0.0390 (5)	0.6669 (2)	0.0568 (7)	
H5	0.4746	0.1334	0.6023	0.068*	
C6	0.5857 (3)	0.0842 (6)	0.7822 (3)	0.0689 (9)	
H6	0.6395	0.2096	0.7945	0.083*	
C7	0.6063 (4)	−0.0521 (7)	0.8776 (3)	0.0781 (11)	
H7	0.6733	−0.0198	0.9548	0.094*	
C8	0.5280 (4)	−0.2371 (8)	0.8595 (3)	0.0839 (11)	
H8	0.5430	−0.3311	0.9245	0.101*	
C9	0.4260 (3)	−0.2863 (6)	0.7449 (3)	0.0683 (9)	
H9	0.3739	−0.4129	0.7339	0.082*	
C10	−0.0414 (3)	0.5221 (5)	0.1919 (2)	0.0523 (7)	
H10	0.0609	0.5847	0.1933	0.063*	
C11	−0.0983 (4)	0.4051 (6)	0.0701 (2)	0.0785 (10)	
H11A	−0.1940	0.3324	0.0691	0.118*	
H11B	−0.0187	0.3080	0.0621	0.118*	
H11C	−0.1179	0.5011	0.0021	0.118*	
C12	−0.1543 (4)	0.6954 (5)	0.1993 (3)	0.0667 (9)	
H12A	−0.2579	0.6394	0.1915	0.100*	
H12B	−0.1583	0.7922	0.1334	0.100*	
H12C	−0.1184	0.7645	0.2778	0.100*	
H1O	0.010 (4)	−0.007 (6)	0.519 (4)	0.076 (12)*	
H1N	−0.107 (4)	0.331 (5)	0.299 (3)	0.068 (10)*	
H2N	0.222 (4)	−0.286 (6)	0.539 (3)	0.075 (11)*	

### Atomic displacement parameters (Å^2^)

**Table:**

	*U*^11^	*U*^22^	*U*^33^	*U*^12^	*U*^13^	*U*^23^
N1	0.0497 (13)	0.0528 (15)	0.0470 (11)	−0.0033 (12)	0.0195 (10)	0.0038 (11)
N2	0.0601 (15)	0.0547 (17)	0.0713 (16)	0.0032 (14)	0.0240 (13)	0.0041 (14)
O1	0.0544 (10)	0.0537 (11)	0.0498 (11)	−0.0009 (10)	0.0186 (8)	0.0034 (10)
C1	0.0639 (16)	0.057 (2)	0.0563 (15)	0.0040 (16)	0.0289 (13)	0.0024 (14)
C2	0.0505 (15)	0.0513 (17)	0.0448 (13)	0.0020 (13)	0.0184 (10)	−0.0016 (13)
C3	0.0598 (17)	0.062 (2)	0.0536 (15)	0.0069 (15)	0.0215 (12)	−0.0056 (14)
C4	0.0434 (14)	0.058 (2)	0.0618 (16)	0.0088 (14)	0.0213 (13)	0.0101 (15)
C5	0.0485 (13)	0.0629 (19)	0.0636 (16)	0.0053 (16)	0.0226 (13)	0.0152 (16)
C6	0.0469 (15)	0.086 (3)	0.0747 (19)	0.0013 (17)	0.0180 (14)	0.005 (2)
C7	0.0604 (19)	0.105 (3)	0.0686 (19)	0.004 (2)	0.0158 (15)	0.006 (2)
C8	0.073 (2)	0.112 (3)	0.069 (2)	0.025 (2)	0.0243 (17)	0.037 (2)
C9	0.0603 (18)	0.064 (2)	0.088 (2)	0.0063 (16)	0.0320 (16)	0.0212 (18)
C10	0.0546 (14)	0.0559 (17)	0.0490 (14)	−0.0103 (15)	0.0181 (11)	0.0042 (14)
C11	0.103 (2)	0.084 (3)	0.0494 (15)	0.000 (2)	0.0210 (15)	0.0005 (17)
C12	0.0734 (19)	0.065 (2)	0.0615 (16)	0.0042 (17)	0.0174 (14)	0.0097 (16)

### Geometric parameters (Å, º)

**Table:**

N1—C1	1.465 (3)	C5—H5	0.9300
N1—C10	1.476 (3)	C6—C7	1.358 (5)
N1—H1N	0.85 (3)	C6—H6	0.9300
N2—C4	1.384 (4)	C7—C8	1.368 (6)
N2—C3	1.440 (4)	C7—H7	0.9300
N2—H2N	0.89 (3)	C8—C9	1.390 (5)
O1—C2	1.419 (3)	C8—H8	0.9300
O1—H1O	0.79 (4)	C9—H9	0.9300
C1—C2	1.514 (3)	C10—C12	1.512 (4)
C1—H1A	0.9700	C10—C11	1.521 (4)
C1—H1B	0.9700	C10—H10	0.9800
C2—C3	1.527 (4)	C11—H11A	0.9600
C2—H2	0.9800	C11—H11B	0.9600
C3—H3A	0.9700	C11—H11C	0.9600
C3—H3B	0.9700	C12—H12A	0.9600
C4—C9	1.391 (4)	C12—H12B	0.9600
C4—C5	1.399 (4)	C12—H12C	0.9600
C5—C6	1.384 (4)		
			
C1—N1—C10	114.0 (2)	C7—C6—C5	120.9 (3)
C1—N1—H1N	110 (2)	C7—C6—H6	119.5
C10—N1—H1N	107 (2)	C5—C6—H6	119.5
C4—N2—C3	124.2 (3)	C6—C7—C8	119.6 (3)
C4—N2—H2N	115.0 (19)	C6—C7—H7	120.2
C3—N2—H2N	114 (2)	C8—C7—H7	120.2
C2—O1—H1O	109 (2)	C7—C8—C9	120.8 (3)
N1—C1—C2	112.8 (2)	C7—C8—H8	119.6
N1—C1—H1A	109.0	C9—C8—H8	119.6
C2—C1—H1A	109.0	C8—C9—C4	120.3 (3)
N1—C1—H1B	109.0	C8—C9—H9	119.8
C2—C1—H1B	109.0	C4—C9—H9	119.8
H1A—C1—H1B	107.8	N1—C10—C12	109.6 (2)
O1—C2—C1	108.4 (2)	N1—C10—C11	113.4 (3)
O1—C2—C3	111.7 (2)	C12—C10—C11	110.7 (2)
C1—C2—C3	108.6 (2)	N1—C10—H10	107.6
O1—C2—H2	109.4	C12—C10—H10	107.6
C1—C2—H2	109.4	C11—C10—H10	107.6
C3—C2—H2	109.4	C10—C11—H11A	109.5
N2—C3—C2	116.9 (2)	C10—C11—H11B	109.5
N2—C3—H3A	108.1	H11A—C11—H11B	109.5
C2—C3—H3A	108.1	C10—C11—H11C	109.5
N2—C3—H3B	108.1	H11A—C11—H11C	109.5
C2—C3—H3B	108.1	H11B—C11—H11C	109.5
H3A—C3—H3B	107.3	C10—C12—H12A	109.5
N2—C4—C9	119.4 (3)	C10—C12—H12B	109.5
N2—C4—C5	122.9 (3)	H12A—C12—H12B	109.5
C9—C4—C5	117.7 (3)	C10—C12—H12C	109.5
C6—C5—C4	120.6 (3)	H12A—C12—H12C	109.5
C6—C5—H5	119.7	H12B—C12—H12C	109.5
C4—C5—H5	119.7		
			
C10—N1—C1—C2	172.8 (2)	C9—C4—C5—C6	−2.0 (4)
N1—C1—C2—O1	53.6 (3)	C4—C5—C6—C7	1.1 (4)
N1—C1—C2—C3	175.0 (2)	C5—C6—C7—C8	0.4 (5)
C4—N2—C3—C2	−76.6 (3)	C6—C7—C8—C9	−0.8 (5)
O1—C2—C3—N2	−57.1 (3)	C7—C8—C9—C4	−0.2 (5)
C1—C2—C3—N2	−176.5 (3)	N2—C4—C9—C8	−178.9 (3)
C3—N2—C4—C9	176.0 (2)	C5—C4—C9—C8	1.6 (4)
C3—N2—C4—C5	−4.5 (4)	C1—N1—C10—C12	−173.3 (2)
N2—C4—C5—C6	178.5 (2)	C1—N1—C10—C11	62.5 (3)

### Hydrogen-bond geometry (Å, º)

**Table:**

*D*—H···*A*	*D*—H	H···*A*	*D*···*A*	*D*—H···*A*
O1—H1*O*···N1^i^	0.79 (4)	2.09 (4)	2.878 (3)	173 (3)
N2—H2*N*···O1^i^	0.89 (3)	2.26 (3)	3.141 (3)	170 (3)

Symmetry code: (i) −*x*, *y*−1/2, −*z*+1.
